# Deep ECG-Respiration Network (DeepER Net) for Recognizing Mental Stress

**DOI:** 10.3390/s19133021

**Published:** 2019-07-09

**Authors:** Wonju Seo, Namho Kim, Sehyeon Kim, Chanhee Lee, Sung-Min Park

**Affiliations:** 1 Department of Creative IT Engineering, Pohang University of Science and Technology (POSTECH), Pohang 37673, Korea; 2 Research Center of ONESOFTDIGM, Pohang 37673, Korea

**Keywords:** mental stress detection, electrocardiogram, respiration, machine learning, deep learning

## Abstract

Unmanaged long-term mental stress in the workplace can lead to serious health problems and reduced productivity. To prevent this, it is important to recognize and relieve mental stress in a timely manner. Here, we propose a novel stress detection algorithm based on end-to-end deep learning using multiple physiological signals, such as electrocardiogram (ECG) and respiration (RESP) signal. To mimic workplace stress in our experiments, we used Stroop and math tasks as stressors, with each stressor being followed by a relaxation task. Herein, we recruited 18 subjects and measured both ECG and RESP signals using Zephyr BioHarness 3.0. After five-fold cross validation, the proposed network performed well, with an average accuracy of 83.9%, an average F1 score of 0.81, and an average area under the receiver operating characteristic (ROC) curve (AUC) of 0.92, demonstrating its superiority over conventional machine learning models. Furthermore, by visualizing the activation of the trained network’s neurons, we found that they were activated by specific ECG and RESP patterns. In conclusion, we successfully validated the feasibility of end-to-end deep learning using multiple physiological signals for recognition of mental stress in the workplace. We believe that this is a promising approach that will help to improve the quality of life of people suffering from long-term work-related mental stress.

## 1. Introduction

Mental health is being recognized as an important issue in the workplace [1]. If mental stress is not treated in a timely manner (i.e., left unmanaged), employees can experience serious physical problems, such as heart disorders, diabetes, cancer, and stomachaches [2,3]. Stress also causes mental disorders such as depression and anger, and can even lead to suicide [2,4]. Such problems can seriously reduce productivity owing to absences and work disability [1], with the medical and socioeconomic costs in the United States adding up to $300 billion annually [5]. Detecting and relieving stress in a timely manner could thus improve overall healthcare substantially.

Stress is typically evaluated using a stress indicator questionnaire, where individuals answer questions such as the perceived stress scale (PSS) [6] and sleep quality assessment (PSQI) [7], and healthcare professionals evaluate the stress score based on those answers. Because these methods rely on expert evaluations, they are not suitable for continuously monitoring stress in the workplace. This limitation makes it difficult or impossible to recognize stress rapidly and intervene appropriately to help people suffering from it. Consequently, there is a growing need for ways to continuously and objectively monitor stress.

The autonomic nervous system comprises the sympathetic nervous system (SNS) and the parasympathetic nervous system (PNS). When an individual is mentally stressed, the PNS activity decreases and the SNS starts to dominate. These neurological changes lead to physiological changes in heart rate (HR), skin conductance, respiration (RESP), and pupil diameter [2] that can be accurately measured by conventional biomedical instruments. Unfortunately, conventional instruments for measuring physiological signals are not optimal for continuous use in the workplace owing to their bulky size and associated cables. However, the recent advancement of wearable technology has made it practical to continuously measure various physiological signals with minimal disturbances, leading to increased research interest in continuous stress detection based on physiological signals.

Similar to the importance of the developments in monitoring devices, developing algorithms to analyze the collected data and accurately recognize the occurrences of stress is also crucial. Several machine learning models have been proposed to recognize stress based on multiple physiological signals [8,9,10,11,12]. Although these models have demonstrated the feasibility of recognizing stress, they have one serious limitation, namely that machine learning approaches require us to extract well-defined, handcrafted features and find the best way to combine them, both very challenging tasks [13]. Furthermore, because the dependence of such approaches on handcrafted features means they cannot find new stress-related features, it can limit their maximum generalization performance. Overcoming this limitation will require a breakthrough.

Recently, deep learning approaches have made great strides in image processing and natural language processing [14]. This is because they not only automatically extract features from data, but also learn new high-level features based on low-level ones owing to their hierarchical structure, something that simple machine learning models cannot do. In particular, convolutional neural networks (CNNs) and long short-term memories (LSTMs) have led to great successes in numerous fields. Owing to these advantages, attempts have also been made to use this approach to recognize stress [5,12,15]. However, these have only considered one type of physiological signal. Because a single signal cannot capture all possible responses to stress, this may degrade their generalization performance. Conversely, the performance degradation can be solved as well as the diversity of individual physiological characteristics be considered using multiple physiological signals. It is thus essential to study the validity and feasibility of deep learning approaches based on multiple physiological signals.

Our goal in this study is to propose an end-to-end deep neural network based on combining two types of physiological signals, namely electrocardiogram (ECG) and RESP data, which have been proposed as meaningful stress-related signals [10]. In addition, we compare the proposed network with conventional machine learning models and visualize the results to see the activation patterns produced by the ECG and RESP signals.

The remainder of this paper is organized as follows. First, in Section 2, we review the literature on both machine learning approaches using multiple physiological signals and deep learning approaches using one type of physiological signal. Then, our experiment’s protocol, a machine learning approach, and a procedure developing the networks will be covered in Section 3. In Section 4, we provide statistical results, evaluate our proposed network, and compare it with the benchmark machine learning models. Finally, in Section 5, we visualize the activation patterns in our network and compare our study with previous ones that have proposed deep learning approaches. Then, we discuss the use of multiple datasets and conclude the paper by discussing potential limitations and future work.

## 2. Related Works

### 2.1. Machine Learning Approaches

Numerous studies have proposed machine learning approaches for recognizing mental stress based on various types of physiological signals [8,9,11,16]. Of these signals, ECGs and photoplethysmograms (PPGs) have been used to extract handcrafted features related to heart activity, such as the HR and HR variability (HRV). In addition to these, other physiological signals have been investigated, such as RESP, electrodermal activity (EDA), galvanic skin response (GSR), pupil diameter, acceleration, electroencephalograms, electromyograms (EMGs), and electrooculograms. Then, with collected physiological signals, developing such machine learning models requires the following main steps: (1) preprocess and de-noise the data with a digital noise filter; (2) extract well-defined features from the multiple physiological signals and find the best feature set; (3) use these features to train a machine learning model; and (4) evaluate the model on a test dataset.

Siramprakas et al. [8] proposed a stress evaluation model using multiple physiological signals such as ECGs and GSR. In this study, a simulated workplace’s stress was considered to replicate workplace stress and signal data were collected. Then, a support vector machine (SVM) was trained and evaluated with either well-defined features or combinations of features. The model was able to recognize stress with greater than 90% accuracy, leading the authors to conclude that HR, HRV, and GSR features in the time and frequency domains were sufficient to accurately detect stress.

In addition to workplace stress, recognizing stress during driving has also been studied. Here, stress is considered to be a risk factor as it can cause aggressive driving behavior and reduced concentration [16]. In [16], the authors developed two main machine learning models, namely an SVM and a K-nearest neighbors (KNN) approach, to identify three distinct stress levels (low, medium, and high). Using Stress Recognition in Automobile Drivers dataset (DRIVERDB) [10] in PHYSIONET, they collected multiple physiological signals including foot GSR, hand GSR, EMGs, ECGs, and RESP, then extracted well-defined features. By finding the feature set that minimized the error rates, the SVM achieved 99% accuracy with a 5-min time window. Their analysis found that selecting the right model, preprocessing steps, and feature set all helped to maximize its generalization performance.

Betti et al. [11] proposed a wearable physiological sensor system for monitoring stress. They conducted Maastricht Acute Stress Tests and collected multiple physiological signals, including ECGs, EDA, and EEGs. After training, the proposed SVM achieved 86.0% accuracy and found correlations between the handcrafted features and the measured cortisol level, which is regarded as a biomarker of stress. By finding these correlations, the study validated the feasibility of monitoring stress with the proposed wearable sensor system.

### 2.2. Deep Learning Approaches

Although deep learning approaches are heavily used in the image processing and natural language processing fields, and a few studies have used them to detect or recognize stress [5,12,15], no study has yet applied this approach to analyzing multiple signals. Researchers have developed deep neural networks using the following main steps: (1) process the physiological signals with a digital noise filter; (2) design a unique deep neural network based on domain knowledge; (3) train the network; and (4) evaluate it on a test dataset.

Cho et al. [15] proposed a promising approach to recognizing stress with a cheap thermal imaging camera. The collected thermal images of people breathing were preprocessed to create spectrum sequences, and then a CNN was used to extract features from these. To increase the number of data points, a sliding window method was used to augment the data. The proposed CNN achieved greater than 80% accuracy on average for classifying the images as stressed or unstressed. Their main contribution is that they were the first to use spectrum sequences taken from thermal images as input.

Hwang et al. [12] presented the Deep ECG Net for recognizing stress based on short-term ECGs (10 s). The authors proposed a 1D CNN with optimized filter size and pooling length that used domain knowledge of ECG PQRST waveforms. The proposed model achieved better performance than conventional machine learning models. Visualizing the process showed that it detected spiky patterns around ECG P waves, meaning that it was able to automatically extract ECG waveform characteristics. Their network achieved about 80% accuracy on average in classifying the data as stressed or unstressed for their two experiments. Their main contribution is showing that optimized networks based on domain knowledge can provide better performance than conventional machine learning approaches and deep neural networks that are designed without domain knowledge.

He et al. [5] developed a ten-layer CNN to detect acute cognitive stress based on short-term ECGs. Here, spectrum information was used to extract consecutive ECG R-peaks for use as input instead of raw ECG data. This study also used a sliding window method to increase the number of data points. Their results showed that the proposed CNN achieved a lower error rate than conventional machine learning models. In addition, the authors found that the meaningful information related to stress lay in the 0.4–20 Hz range by visualizing the activation maps of multiple layers. This demonstrates that deep learning approaches can benefit from having data-driven features that are not used by conventional machine learning approaches.

## 3. Methods

### 3.1. Subjects

We recruited 18 subjects (8 females and 10 males, aged 24.6 ± 4.6) from Pohang University of Science and Technology (POSTECH), South Korea, via open recruiting. The study subjects were selected based on the following criteria: (1) they had no cardiovascular disease or mental problems and (2) they had not undertaken intense exercise before the day of the test and (3) they had not had caffeinated beverages on the day of the test. This study was approved by the POSTECH Ethics Committee (PIRB-2019-E001).

The experiments were conducted in parallel with the recruiting process. Although most experiments proceeded normally, unexpected technical problems (namely a subject’s carelessness and an unexpected Windows OS update) occurred during two experiments, meaning that these two subjects’ data were not captured correctly. Thus, we only considered the datasets collected from the remaining 16 subjects.

### 3.2. Experiments

#### 3.2.1. Protocol

**Figure 1:**
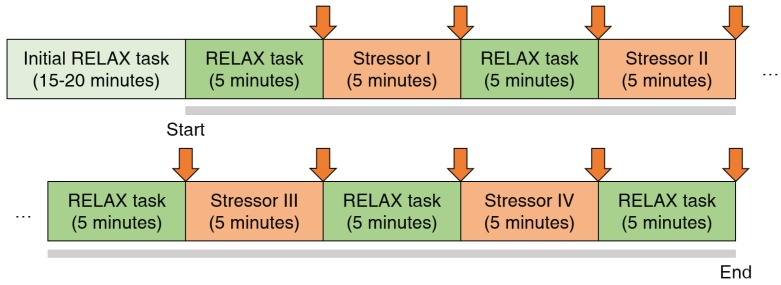
An experiment process. There are two stages for the whole experiment: an initial relaxation stage (colored with light green), and a regular experiment stage (indicated by gray bold lines). The regular experiment stage can be segmented into 5 min of relax tasks (colored with dark green) and stress tasks (colored with dark orange). From the start to end of the regular experiment stage, subjects’ physiological signals were captured by a wearable device. At the end of each short a relax or a stress task, a mental stress level assessment was carried (indicated with an orange arrow).

Each experiment set took about 1 h, and comprised two stages: (1) an initial relaxation stage before the experiment began (about 15–20 min) and (2) the main experimental stage (about 45 min). These are described in more detail in Figure 1.

During the initial relaxation stage, each subject was asked to wear a wearable device that collected ECG and RESP data and to completely relax, eliminating any excitement or nervousness regarding the experiment. In addition, we explained our protocol in detail to prevent any mistakes by the subject. During the main experimental stage, the subjects alternately experienced simulated relaxing states (called RELAX tasks in Figure 1) and stressful states. During the relaxation tasks, the subject was asked to sit on a chair in a comfortable position without any mental activity. The first relaxation task aims to build a psychological baseline and remove unwanted excitement before the regular experiment stage. Similarly, the other relaxation tasks aim to remove stress after a stressful task and prepare the next task by setting the psychological baseline. This design improves the reliability of the experiment’s results [5]. During the stressful tasks, the subject was provided with one of two types of stressors: (1) a math task, namely a quiz requiring the subject to solve a series of subtraction problems via mental arithmetic, or (2) a Stroop task, namely a quiz where the subject was asked to respond with the color of a given word and ignore its meaning. Because all the subjects were Korean, the words were presented in the Korean language. These are typical tasks that have been used to induce stress in previous studies [12,15,17].

The tasks also varied in difficulty, based on the results of a previous study by Cho et al. [15]. For example, an easy math task might be to repeatedly subtract 1 from a four-digit number, responding within 7.5 s, while a hard math task might involve repeatedly subtracting a two-digit number, rather than 1, from a four-digit number with the same time limit. Likewise, easy Stroop tasks involved words with the same color and meaning, while these were mismatched for hard Stroop tasks. In either case, the time limit for each Stroop problem was 1.5 s. Appropriate sound feedback was also provided to indicate whether or not the entered answer was correct, encouraging the subject to pay attention to the task and inducing additional stress.

The subjects were presented with these four stress-inducing tasks (two types with two difficulties) in random order. Although a previous study [15] used a fixed order, we chose not to do this, for two reasons: (1) a fixed order could bias the stress level, and (2) a random order would better replicate real stress-inducing situations. All of the relaxation and stressor tasks lasted for 5 min. At the end of each task, the subject was asked to evaluate how mentally stressed he or she felt, based on a visual analogue scale (VAS) [15], which is used as a evaluation method of an individual’s subjective stress score with value from 0 (not at all) to 10 (extreme stress). For example, if a subject is relaxed or stressed, the VAS score will be close to zero or 10, respectively. In this study, the purpose of the use of VAS is to confirm the average effects (e.g., induce stress or relieve stress) of each task on the 16 subjects. During the experimental stage, the subjects were asked not to use their cellphones and to minimize unexpected mental stimuli. When the experimental stage was complete, the subject took off the wearable device to complete the experiment.

#### 3.2.2. Experimental Setup

A BioHarness module 3.0 (Zephyr Technology, Annapolis, MD, USA) was used to collect the subjects’ ECG and RESP data. This wearable device is compact and can be tightened with a strap, making it a good choice to minimize movement disturbance during the experiment. Because making the strap too tight could induce unnecessary stress or pain, we asked the subjects not to tighten it so much that it was uncomfortable to wear.

**Figure 2:**
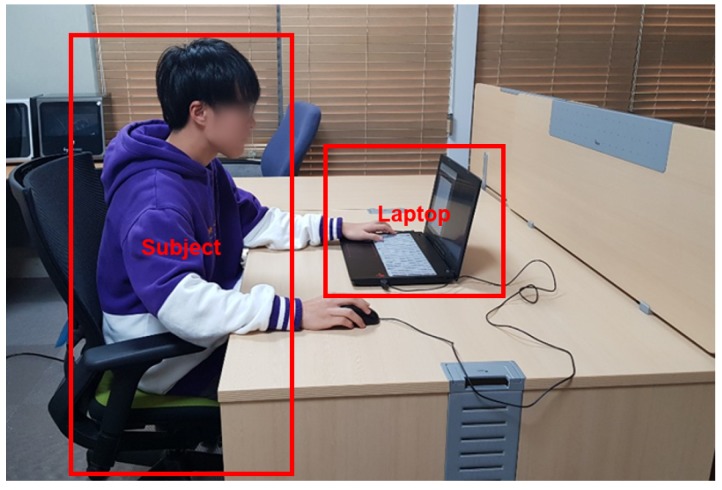
Setup of the experiment in a closed room. A subject proceeds with the experiment with a laptop computer. There was not only no one else except the subject, but also no camera not to make the subject nervous or embarrassed.

The experiments were conducted on a laptop computer (with a 2.8 GHz Intel Core i7 processor (Santa Clara, CA, USA) and 16 GB of RAM) in a closed room. During each experiment, the subject was alone in the room, as shown in Figure 2. On the laptop computer, a graphical user interface application was installed, which we developed with MATLAB R2016a (MathWorks, Natick, MA, USA). This was designed to be as simple as possible so as not to confuse the subjects.

#### 3.2.3. Data Preprocessing

After running the experiment a total of 16 times, we collected 16 datasets, consisting of ECG and RESP data, and stress level indices (VAS scores).

During the preprocessing step, the captured ECG signal was first filtered by a 2000th-order finite impulse response notch filter with 58–62 Hz bandwidth, and a second by a 3000th-order finite impulse response bandpass filter with 1.5–150 Hz bandwidth [12]. This de-noising process makes it easy to find the R-peaks of ECG. In contrast, during the preprocessing of the RESP signal, we did not filter this because it was captured from torso expansion and contraction, and thus any motion noise might not be independent of the subject’s breathing.

We divided the segment for each task into six clips, consisting of 50-s windows with no overlap. We chose 50-s windows because at least 50 s of physiological data are required to extract several important features [17]. The ECG and RESP segments’ start and end times were all clearly synchronized. Here, there was only one data point of overlap between one segment and the next. Then, we excluded the first clip from each segment owing to the initialization time needed for each task. After preprocessing, we obtained a total of 720 segments (16 subjects, each recording nine segments, with five clips per segment). Finally, we labeled each segment with its binary class (stressed or unstressed) according to the task type (relaxation or stressor).

### 3.3. Machine Learning Approaches

To compare our deep learning approach with conventional machine learning approaches, we also developed several machine learning models for use as benchmarks. Here, we selected ECG and RESP features that have been used in many previous studies [11,12,17,18,19].

We extracted 11 handcrafted features from the ECG data, including four time-domain features and seven frequency-domain features (Table 1). As time-domain features, we extracted the mean HR (HR mean), standard deviation of the Normal-to-Normal (NN) interval (sdNN), root mean square of successive difference of R peak-to-R peak (RR) intervals (rmssd), and percentage of the differences between adjacent RR intervals that were greater than 50 ms (pNN50). As frequency-domain features, we extracted the NN interval powers in the following ranges: 0.00–0.04 Hz (VLF), 0.04–0.15 Hz (LF), 0.15–0.40 Hz (HF), and 0.14–0.40 Hz (TF). In addition, we included the ratios of LF to LF+HF (nLF), HF to LF+HF (nHF), and LF to HF (LF2HF) as frequency-domain features.

We also extracted a total of eight handcrafted RESP features: three time-domain features and five frequency-domain features (Table 1). As time-domain features, we used the square root of the mean squared RESP (RMS), interquartile range (IQR), and mean difference between adjacent elements of each RESP segment (MDA). As frequency-domain features, we used the powers in the 0.00–1.00 Hz (LF1), 1.00–2.00 Hz (LF2), 2.00–3.00 Hz (HF1), and 3.00–4.00 Hz (HF2) ranges, as well as the LF1 + LF2 to HF1 + HF2 ratio (L2H). As with the ECG frequency-domain features, the RESP features were also computed using Welch’s method of estimating the data’s power spectral density.

Then, we developed several machine learning models that have previously been proposed to classify stress states [20]. While the models were being trained and evaluated, the features were normalized by using a MinMax scaler to bring them into the 0–1 range. To prevent data leakage during training, the scaler parameters were fitted using only the training set features, but used to normalize both the training and test set features. We tuned the models’ hyper-parameters via grid search and calculated their average performance using five-fold cross validation.

### 3.4. Deep Learning Approaches

Unlike machine learning approaches, deep learning approaches are based on deep neural networks that can directly extract features from the data, and are not reliant on well-defined handcrafted features. As the name implies, deep neural networks are artificial neural networks with two or more hidden layers. Having many hidden layers enables such networks to learn more complex nonlinear patterns and hierarchical information than would be possible with shallow networks. Despite these advantages, however, deep neural networks also usually have a large number of parameters, which can lead to over-fitting, and they can experience issues with the gradient vanishing when they have a large number of layers. These problems can result in a failure to learn and an increase in generalization errors. To overcome these limitations, recent algorithmic advances (e.g., rectified linear units, batch normalization, dropout, stochastic gradient descent, and data augmentation), more powerful computational hardware (e.g., general-purpose graphical processor units), and innovative network architectures, such as CNNs and LSTMs, have partially resolved these over-fitting and gradient vanishing problems, enabling high performance to be achieved. These developments have encouraged the use of deep learning approaches in numerous fields, including physiological signal analysis [21] and stress recognition [5,12,15].

**Figure 3:**
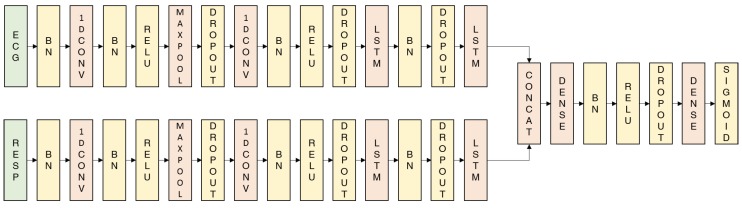
The structure of the proposed DeepER Net. The different signals were processed in each network branch and then concatenated for recognizing the stress. The basic structure is based on the structure of Deep ECG Net [12].

We designed our proposed network based on Deep ECG Net’s structure [12]. First, a batch-normalization layer is used to normalize each physiological signal, so that the network can learn to normalize the signals based on the data itself. Then, there is a 1D convolutional layer and a 1D max-pooling layer for each signal, which extract stress-related waveform patterns from the ECG and RESP data. Here, a rectified linear unit (ReLU) is used as the activation function. Next, comes another 1D convolutional layer. There is no additional max-pooling layer this time because the previous max-pooling process has greatly reduced the dimensionality. After that, there are multiple LSTM layers, in order to obtain sequential information about the features extracted from the previous convolutional layer. Next, we concatenate the extracted ECG and RESP features and add a dense layer. Finally, there is a fully-connected layer with a sigmoid activation function, which classifies the data as stressed or unstressed. To prevent over-fitting, we also add dropout and batch-normalization layers. Figure 3 shows the structure of the proposed DeepER (ECG–RESP) Net.

As noted by the developer of Deep ECG Net [12], both the first 1D convolutional layer’s kernel length and 1D max-pooling layer’s pooling length are important factors. They determined that a kernel length of 0.6 s (i.e., 600 points at a sampling frequency of 1 kHz) and a pooling length of 0.8 s (i.e., 800 points) were optimal. These choices are very plausible. First, the length of the PQRST of the ECG is the sum of its PR and QT intervals that is between 0.57 and 0.67 [12]. Thus, selecting a value between them is reasonable as a kernel length. Furthermore, to apply a max-pooling operation of an interval including at least one R peak that is related to HR and HRV, an average heart rate period (about 0.8 s) can be a considerable candidate. Based on these heuristic choices, we designed our first 1D convolutional layer to have the same kernel and max-pooling lengths (0.6 s and 0.8 s, respectively) for processing the ECG data. The kernel and max-pooling lengths of the network designed to process RESP data were designed similarly: a single respiration period was used for the kernel and max-pooling lengths. Because the RESP pattern is simple and split into by an expiration (e.g., nadir) and an inspiration (e.g., peak), the size is sufficient to extract RESP’s features. Because adults normally respire 12–20 times per min [22], we set both lengths to 5 s (i.e., 125 points at a sampling frequency of 25 Hz).

Our proposed network has 50 filters in each of the initial 1D convolutional layers, which has a stride of 1. For the ECG network, there are 50 filters in the second 1D convolutional layer, which has a kernel length of 25 and a stride of 1. For the RESP network, there are 50 filters in the second 1D CNN layer, which has a kernel length of 4 and a stride of 1. Zero-padding was used in all the convolutional layers to maintain the input size. There are 32 and 16 units in the first and second LSTM layers, respectively, and 512 units in the dense layer. The second 1D convolutional layers in the ECG and RESP networks have kernel lengths of 25 and 4, respectively, so as to focus on the same time interval (20 s). All dropout layers have a dropout rate of 0.5 and the weight decay’s regularization strength is $10^{-4}$.

For training, we used the Adam [23] optimizer with a learning rate of $10^{-3}$ and a step decay scheduler (i.e., the learning rate is halved every 50 epochs). The binary cross-entropy loss was used to calculate the losses between the labels and predictions, as follows:

(1) $$L=-\frac{1}{M}\sum_{i=1}^{M}y_{i}\log\left(\hat{y_{i}}\right)+\left(1-y_{i}\right)\log\left(1-\hat{y_{i}}\right).$$

We used a total of 250 epochs, a batch size of 32, and a 0.3 validation split (i.e., 30% of the training set). Finally, the model with the lowest loss on the validation set after 250 epochs was used for evaluation. As with the machine learning models, we used five-fold cross validation to evaluate the performance of the network.

All training processes were conducted using the well-known Keras deep learning library, with Python 2.7 running under Ubuntu 16.01.5, on a PC with a 3.6 GHz Intel Core i7 processor, 128 GB of RAM, and 4 NVIDIA GeForce GTX1080 Ti (Santa Clara, CA, USA).

### 3.5. Metrics

Because this is a binary classification problem (i.e., the subject is stressed or unstressed), we used the following metrics to evaluate both the deep learning network and the machine learning models:

(2) $$Accuracy=\frac{TP+TN}{TP+TN+FP+FN}\times 100\;\left(\%\right),$$

(3) $$F1\;\mathrm{score}=2\times \frac{TP}{2\times TP+FP+FN}.$$

Here, TP (true positive) is the number of cases correctly classified as “stressed,” while TN (true negative) is the number of cases correctly classified as “unstressed.” Likewise, FP (false positive) is the number of cases that were classified as “stressed” but were actually “unstressed,” while FN (false negative) is the number of cases that were classified as “unstressed” but were actually “stressed.” The first metric (accuracy) is the percentage of cases that were correctly predicted, while the second (F1 score) is the harmonic mean of the precision and recall, which indicates the trade-off between these two metrics.

In addition to the accuracy and F1 score, we also used the area under the receiver operating characteristic (ROC) curve to evaluate the models. The area under the ROC curve (AUC) is a well-known model accuracy metric [24]. By calculating each sensitivity and specificity according to probability thresholds, which is within 0 to 1, the ROC is independent of the different thresholds and thus the metric is reliable and reflects the average performance with the thresholds. Models with AUCs above 0.9 are considered to be accurate [24].

## 4. Results

In our experiments, we collected a total of 144 VAS scores for individual tasks from 16 subjects. These were evaluated after each relaxation and stressor task (Figure 1). To eliminate any inter-subject variability, the scores were normalized for each subject. Table 2 shows the average normalized scores.

As Table 2 shows, the scores are significantly lower for the relaxation tasks than for the stressor ones. Among the stressor tasks, the hard and easy math tasks yielded the highest and lowest average scores, respectively. Contrary to our expectations, the average score was lower for the hard Stroop task than for the easy one, possibly because easy but tedious tasks may be more stressful than difficult tasks. However, if the task is too difficult, as with the hard math task, it appears to be more stressful than a tedious task.

Because our experiments involved alternating relaxation and stressor tasks, we also calculated the average difference between the normalized VAS score recorded immediately before a stressor task (i.e., after a relaxation task) and that recorded immediately after the stressor (Table 3). Here, it is clear that all the stressor tasks induced stress, and that the most and least stressful tasks were the hard and easy math tasks, respectively, as in Table 2. Again, the easy Stroop task was a stronger stressor than the hard one.

### 4.1. Performance

Among the 720 segments, one of the ECG segments was significantly distorted by a motion noise; we excluded this segment and its label for further analysis. To evaluate the performance of a model, five-fold cross validation was used on both machine learning models and DeepER Net. This method commonly evaluates the predictability of a model [5]. In particular, the 719 segments were randomly shuffled and split into five folds. Furthermore, five-fold cross validation was applied for evaluation. The use of this cross validation scheme is independent of subjects, indicating that the segments extracted from a subject can be in both test set and training set. Because of the similarity between the test set and training set, this might lead to higher accuracy on the testing set.

We calculated the average performance of the machine learning models, as shown in Table 4, and then selected the best model for comparison purposes. Of these models, the random forest (RF) yielded the highest average accuracy (71.8 ± 2.3%), F1 score (0.67 ± 0.04), and AUC (0.80 ± 0.02). This was followed by the decision tree (DT), then SVM, the KNN, and finally the logistic regression (LR) showed the lowest performance. This highlights the fact that different models can give different performance, even when trained on the same handcrafted feature set, and that we need to find the most suitable model for each problem. In addition, the fact that the RF and LR demonstrated the highest and lowest performance, respectively, suggests that an ensemble model can be suitable for recognizing stress. However, the RF’s AUC was less than 0.9, so it cannot be considered to be highly accurate [24].

Turning now to the performance of the proposed DeepER Net, we find that it showed the highest average accuracy (83.9 ± 2.3%), F1 score (0.81 ± 0.05), and AUC (0.92 ± 0.01). Compared with the RF, its average accuracy was 12.1% higher (*p*-value < 0.05 with paired *t*-test), its average F1 score was 0.14 higher (*p*-value < 0.05 with paired *t*-test), and its average AUC was 0.12 higher (*p*-value < 0.05 with paired *t*-test), clearly indicating that our deep learning approach was a substantial improvement. In addition, DeepER Net’s AUC was greater than 0.9, so we can conclude that it is highly accurate for recognizing stress [24]. These results thus suggest that our deep learning approach is a promising option for accurately recognizing stress. Loss and accuracy information of the proposed DeepER Net during training is shown in Figure S1.

### 4.2. Visualization

**Figure 4:**
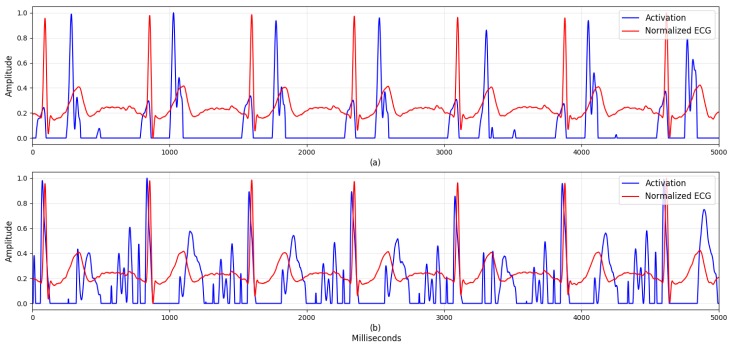
The activations on the first ReLU of electrocardiogram (ECG) signal. To easily see which signal patterns were activated, the activations and the first batch-normalization layer’s output were normalized with MinMax Scaler having a range from 0 to 1. The blue line indicates the activations and the red line indicates the output. Activations around (**a**) ECG Q and T’s ascending waveform and (**b**) ECG QRS and T’s descending waveform.

**Figure 5:**
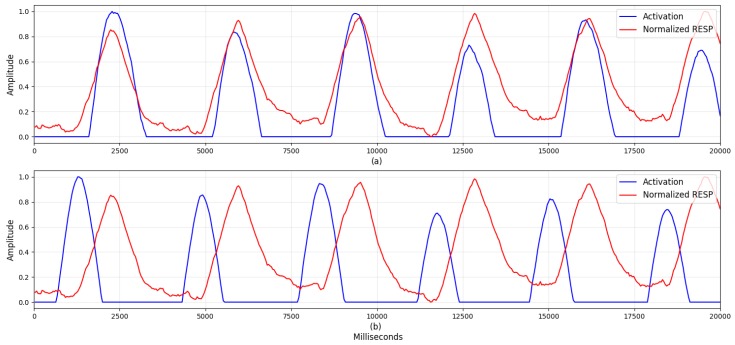
The activations on the first ReLU of respiration (RESP) signal. To easily see which signal patterns were activated, the activations and the first batch-normalization layer’s outputs were normalized with MinMax Scaler having a range from 0 to 1. The blue line indicates the activations and the red line indicates the output. Activations around (**a**) RESP peak (e.g., inspiration) and (**b**) RESP nadir (e.g., expiration).

Although numerous studies have considered machine learning models based on handcrafted features, Table 4 shows that our deep learning approach provided superior performance. This suggests that data-driven features can capture more general information than handcrafted ones. Visualizing the neurons’ activation is a potentially useful way to further analyze these results, as it can help researchers to understand how the network is making its decisions and find new stress-related features. Here, we selected the network trained during the first fold of cross validation and a sample of the ECG and RESP data. Then, after calculating the activation in both parts of the network, we compared the first batch-normalization layer’s output with the activation after the first ReLU for each signal. Because we used zero-padding in the convolutional layer to maintain the input length, we also applied zero-padding to the first batch-normalization layer’s output. The activations of the ECG and RESP networks are shown in Figure 4 and Figure 5, respectively.

Figure 4 shows how the neurons in the proposed Deep ER Net were activated by periodic and comprehensive ECG waveform patterns, for (a) Q and T’s ascending waveform, and (b) QRS and T’s descending waveform. These results indicate that the filters were able to extract these unique ECG waveforms, unlike the machine learning approaches considering only ECG’s R-peaks. In Figure 5, we find that neurons were activated around the RESP peaks and troughs. This is clear because their periodic patterns are closely related to stressed or relaxed states. These results show specific patterns, including peaks, troughs, and waveforms, from which we can conclude that the proposed DeepER Net was making decisions based on information about the periods of specific ECG and RESP patterns.

## 5. Discussion

### 5.1. Visualization

Visualization is a promising approach to finding evidence for how networks make decisions. Of the various visualization tools, we elected to look at the activation of the network’s neurons to identify which ECG and RESP patterns they focused on. As Figure 4 shows, the neurons were activated around ECG QRS, and T waveforms. These are unique patterns, specific to ECG data, and the network’s convolutional layers were able to consider changes in their shape and amplitude. Likewise, Figure 5 shows that the network was able to process patterns extracted around the RESP peaks and troughs.

These findings indicate that our network can extract a more comprehensive range of features than simple handcrafted ones that consider specific waveform (e.g., R-peaks), frequency-domain, or time-domain features. This is possible because the network learned meaningful stress-related features from the data. From this point of view, we can understand why the network performed better than the machine learning models (Table 4). We can therefore conclude that this deep learning approach is more promising than the previously proposed machine learning approaches.

### 5.2. Comparison with Previous Studies

Three studies [5,12,15] have proposed deep learning approaches to stress recognition. Deep ECG Net [12]’s structure was optimized using domain knowledge about the ECG PQRST waveforms, enabling it to achieve a high average accuracy of 80.7% on two different datasets and perform better than conventional machine learning models. Consequently, we used this optimized network structure as the basis for our proposed DeepER Net. Next, because good experimental protocol design is important for obtaining reliable datasets and results, we adapted Cho et al.’s [15] well-designed protocol for use in our study. They proposed a cheap thermal imaging-based stress detection method, which extracts multiple spectrum images from the thermal respiration images and then augments the data using a sliding window method. The resulting CNN achieved 84.6% accuracy for classifying two stress levels (binary classification). Finally, He et al. [5] proposed a deep CNN for detecting acute cognitive stress from 10-s ECGs. They used spectrum images extracted around ECG R-peaks as input and applied data augmentation. Their CNN achieved an average error rate of 17.3%, equivalent to an average accuracy of 82.7%.

In this study, we have proposed the first end-to-end deep neural network (DeepER Net) to recognize stress using multiple signals (ECG and RESP). Because we needed to consider two different signals, we developed a unique network structure that could extract features from both signals. The network achieved an average accuracy of 83.9%, which is comparable to the results achieved by the other proposed models [5,12,15] as summarized in Table 5. For a fair comparison, evaluating the models on a public dataset via the same training conditions and evaluation method can be useful. We proceeded with an experiment validating the models using the DRIVERDB [10] including ECG, RESP, and stress label information. The dataset [10] was collected with the different driving sections (e.g., rest, city, and highway) and each section indicates different stress level. For example, the rest section, city section, and highway section indicate low, high, and medium stress levels, respectively. Among a 17 drivers dataset in [10], we considered only 11 drivers having an existence of the clear marker [25]. The preprocessing including noise filtering and clipping was the same presented in the Methods section. After preprocessing, 801 labeled segments including ECG, RESP, and Lomb Periodogram spectrum [5] were obtained. Finally, the last layer of networks was replaced with a softmax layer for classifying three classes (e.g., low, medium, and high) and then we trained and evaluated the three networks with five-fold cross validation on the segments. As a result, the proposed DeepER Net showed the highest average accuracy of 83.0%; the Deep ECG Net [12] showed the average accuracy of 75.0% and the network [5] showed the average accuracy of 38.5% which may be owing to under-fitting caused by the small capacity of the network. This result means that the use of the multi physiological signals improves the performance of recognizing stress. However, we guess that there may be performance degradation in the open dataset because several important hyper-parameters of networks have been optimized in their dataset, not the open dataset. Thus, more open and reliable data needs to be disclosed. The hyper-parameters, learning rule, and structure of networks [5,12] are shown in Tables S1 and S2.

By visualization, we also identified the activation patterns produced by the ECG and RESP data and analyzed their meanings. Although previous studies have analyzed ECG activation patterns [5,12], ours is the first to analyze the various ECG and RESP activation patterns related to stress recognition, which we believe makes it distinctly different from previous work.

### 5.3. Possibility of Personalized Models

Although this study did not focus on personalized models that can adapt to individual stress responses, such models could be developed based on the proposed network. Because DeepER Net’s last layer is a sigmoid function, the probability of stress is calculated within a 0–1 range and the model then makes a decision using the default threshold (0.5). Increasing the threshold would make the model stricter when determining stress states, while lowering the threshold would make it more generous. This suggests that we could change the threshold based on individual stress responses, and hence develop personalized models. Alternatively, personalized models could be developed by fine-tuning the network based on data from a single individual. Unlike with conventional machine learning approaches, there is no need to retrain the network from scratch, so it can be trained rapidly and avoid over-fitting issues.

### 5.4. Multiple Physiological Datasets

The main reasons for using multiple physiological datasets are as follows. First, a small number of subjects can cause over-fitting problems that reduce generalization performance. Such over-fitting issues can be overcome by increasing the amount of data (e.g., by involving more subjects or augmenting the data) or using features based on other independent types of data. Because increasing the number of subjects is difficult, extracting independent features can help to deal with over-fitting problems. In addition, each person’s stress responses may vary slightly, leading to the problem of inter-variability, which has the effect of lowering generalization performance for new subjects. Therefore, considering multiple data related to stress could help to reduce the problem.

However, using too many different types of data could reduce the stress recognition system’s usability by requiring a variety of monitoring devices to be worn to collect all the different physiological signals, which would be burdensome in practice. Researchers should thus consider the trade-offs involved between usability and performance.

### 5.5. Limitations and Future Work

Our study has two main limitations: the experimental setting and the use of a respiration monitoring device. Although the setting was intended to simulate a real workplace, the actual experiments were conducted in a more controlled manner because recruiting working subjects is difficult and an uncontrolled experimental setting would have reduced the quality of the data. Once we have established our model’s validity, we plan to perform experiments in a real workplace setting. In this study, we used a chest strap-based wearable device to measure the physiological signals, but we are aware that such devices can be hard to wear in the workplace and thus plan to use a patch-type ECG device and a wearable device to measure RESP in a later study.

## 6. Conclusions

In this study, we have proposed the first end-to-end deep learning approach to stress recognition based on ECG and RESP data. Our protocol involved collecting ECG and RESP data and recording subjective stress scores while the subjects conducted alternating stressor and relaxation tasks. Using this multiple dataset, our proposed DeepER Net performed better than conventional machine learning models that require the extraction of handcrafted features. By visualizing the network’s activation, we found that its neurons were being activated by unique and specific patterns. In conclusion, we believe that our proposed DeepER Net will be of benefit to people who suffer from stress in the workplace.

## Figures and Tables

**Table 1:** A list of features extracted from ECG and RESP. We computed the power spectral density of ECG’s NN interval and RESP, using Welch’s method, to extract frequency domain features. Abbreviations: ECG, electrocardiogram; RESP, respiration; NN, normal-to-normal; RR, R peak-to-R peak.

Signal	Domain	Features	Description
ECG	Time	HR mean	Mean of heartrate
		sdNN	Standard deviation of NN intervals
		rmssd	Root mean square of successive difference of RR intervals
		pNN50	Percentage of differences between adjacent RR intervals that are greater than 50 ms
ECG	Frequency	VLF	Power of NN interval (0.00–0.04 Hz)
		LF	Power of NN interval (0.04–0.15 Hz)
		HF	Power of NN interval (0.15–0.40 Hz)
		TF	Power of NN interval (0.14–0.40 Hz)
		nLF	LF to (LF + HF) ratio
		nHF	HF to (LF + HF) ratio
		LF2HF	LF to HF ratio
RESP	Time	RMS	Square root of mean of squared RESP
		IQR	Interquartile range of RESP
		MDA	Square root of mean of squared differences between adjacent elements
RESP	Frequency	LF1	Power of RESP (0.00–1.00 Hz)
		LF2	Power of RESP (1.00–2.00 Hz)
		HF1	Power of RESP (2.00–3.00 Hz)
		HF2	Power of RESP (3.00–4.00 Hz)
		L2H	(LF1+LF2) to (HF1 + HF2) ratio

**Table 2:** Average normalized visual analogue scale (VAS) scores for all tasks. These have been normalized to a range of 0–1 with a MinMax scaler.

Task	Average Value
Relax	0.24
Easy math	0.51
Easy stroop	0.61
Hard math	0.80
Hard stroop	0.52

**Table 3:** Average differences between the normalized VAS scores before and after each task. Here, the relaxation tasks were used as a baseline before stressor tasks.

Task	Average Value
Easy math	0.12
Easy Stroop	0.42
Hard math	0.55
Hard Stroop	0.32

**Table 4:** Average metrics after five-fold cross validation. We used Equations (2) and (3) to calculate the average accuracy, F1 score, and AUC, as well as their standard deviations, and show these results as average ± standard deviation. Abbreviations: SVM, support vector machine; RF, random forest; KNN, k-nearest neighbors; LR, logistic regression; DT, decision tree; AUC, area under the ROC curve; ROC, receiver operating characteristic.

Model	Accuracy (%)	F1 Score	AUC
DeepER Net	83.9 ± 2.3	0.81 ± 0.05	0.92 ± 0.01
SVM	61.7 ± 3.4	0.62 ± 0.04	0.68 ± 0.05
RF	71.8 ± 2.3	0.67 ± 0.04	0.80 ± 0.02
KNN	64.0 ± 3.2	0.60 ± 0.02	0.67 ± 0.04
LR	59.1 ± 2.5	0.55 ± 0.05	0.63 ± 0.04
DT	68.8 ± 1.6	0.66 ± 0.02	0.70 ± 0.02

**Table 5:** Comparison with the-state-of-the-art deep learning approaches using physiological signals for recognizing stress. Abbreviations: CNN, convolutional neural network; LSTM, long short-term memory.

Models	Physiological Signal	Model	Accuracy
Hwang et al. [12]	ECG	CNN and LSTM	80.7%
Cho et al. [15]	Thermal respiration images	CNN	84.6%
He et al. [5]	Lomb Periodogram spectrum extracted from zero-one transformed NN intervals	CNN	82.7%
Proposed DeepER Net	ECG and RESP	CNN and LSTM	83.9%

## Supplementary Materials

The following are available online at https://www.mdpi.com/1424-8220/19/13/3021/s1. Figure S1: Loss and accuracy information of the proposed DeepER Net during training, Table S1: The structure of the network [12] and its training condition, Table S2: The structure of the network [5] and its training condition.
