# Supplementary material for: Deep ECG-Respiration Network (DeepER Net) for Recognizing Mental Stress
Source: Sensors (Basel). 2019 Jul 9;19(13):3021. doi: 10.3390/s19133021 (PMC6652136; doi:10.3390/s19133021)
Supplement: Supplementary file 1 [file sensors-19-03021-s001.pdf]

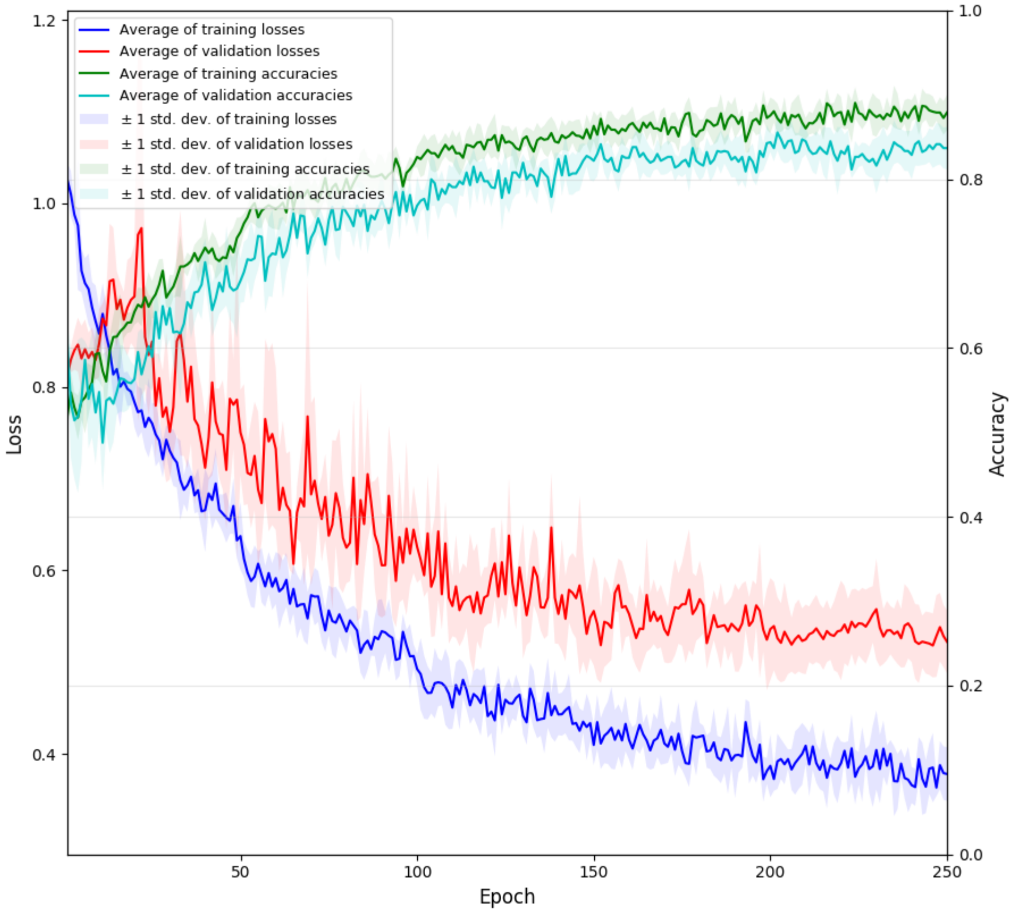

Figure S1. Loss and accuracy information of the proposed DeepER Net during training. The blue line indicates the average of training losses and its transparent box indicates the ± 1 standard deviation of the losses. The red line indicates the average of validation losses and its transparent box indicates the ± 1 standard deviation of the losses. The green line indicates the average of training accuracies and its transparent box indicates the ± 1 standard deviation of the accuracies. The cyan line indicates the average of validation accuracies and its transparent box indicates the ± 1 standard deviation of the accuracies.

Table S1. The structure of the network [12] and its training condition. Adadelta with 1.0 learning rate was used to train the network with 200 epochs and 32 batch size. The input size is determined by 50 s (e.g., 496 $\times$ 50 = 24,800). The kernel size is determined by 0.6 s (e.g., 496 $\times$ 0.6 $\approx$ 300) and the pooling size is determined by 0.8 s (e.g., 496 $\times$ 0.8 $\approx$ 400). Because some hyper-parameters were not stated in [12], the hyper-parameters were set the same using the proposed DeepER Net. The name of hyper-parameters is defined in Keras library

Depth	Layer	Options and hyper-parameters
1	Input layer	Input size = (24800,1)
2	1D convolutional layer	Kernel size = 300, activation = linear, number of filters = 50, stride = 1 kernel intializer = he-normal, padding ='same', kernel regularizer = l2(1e-4)
3	ReLU	
4	1D max-pooling	Pooling size = 400
5	Dropout	Rate = 0.5
6	Batch normalization	
7	LSTM	Units = 32, return sequences = True, kernel initializer = glorot-normal, kernel regularizer = l2(1e-4)
8	Batch normalization	
9	LSTM	Units = 16, return sequences = False, kernel initializer = glorot-normal, kernel regularizer = l2(1e-4)
10	Dense	Units = 3, activation = softmax, kernel regularizer = l2(1e-4)

Table S2. The structure of the network [5] and its training condition. Stochastic gradient descent with 0.001 learning rate and 0.9 momentum was used to train the network with 200 epochs and 1 batch size. The learning rate is divided by 10 every 10 epochs [5]. Because we followed the preprocessing method in [5] and the Lomb Periodogram spectrum (0.04 Hz - 20 Hz) was extracted from the zero-one transformed sequence of 50-s ECG that includes 24,800 sample points (e.g., 496 $\times$ 50), the input size is the same in [5]. This study did not present some of hyper-parameters and then the hyper-parameters were set the same using the proposed DeepER Net. The total number of layer is 10 owing to the last layer, which includes a softmax and an output layer.

Depth	Layer	Options and hyper-parameters
1	Input layer	Input size = (799,1,1)
2	2D convolutional layer	Kernel size = (4,1), activation = linear, number of filters = 6, stride = 1, kernel intializer = he-normal, padding ='valid', kernel regularizer = l2(1e-4)
3	Batch normalization	
4	ReLU	
5	Dropout	Rate = 0.5
6	Dense	Units = 10, activation = linear, kernel regularizer =l2(1e-4)
7	Batch normalization	
8	Dense	Units = 3, activation = softmax, kernel regularizer = l2(1e-4)
